# A scoping review on social isolation in patients with intestinal stomas

**DOI:** 10.3389/fpsyg.2026.1919034

**Published:** 2026-08-19

**Authors:** Xiaoyan Zhang, Jihong Yang, Aihong Gao, Yujing Chen

**Affiliations:** 1 School of Nursing, Shanxi University of Chinese Medicine, Jinzhong, Shanxi, China; 2 Graduate School, Shanxi University of Chinese Medicine, Jinzhong, Shanxi, China; 3 The Second Department of Anorectal, Affiliated Hospital of Shanxi University of Chinese Medicine, Taiyuan, Shanxi, China

**Keywords:** evidence-based nursing, influencing factors, intestinal stoma, scoping review, social alienation

## Abstract

**Objective:**

Systematically sort out the psychological experiences, influencing factors, and intervention measures of social isolation in patients with intestinal stomas, providing evidence-based support for subsequent research.

**Methods:**

Search for relevant studies on social isolation in patients with enterostomy in CNKI, Wanfang Database, VIP Database, China Biomedical Literature Database, PubMed, Web of Science, Embase, Cochrane Library, and CINAHL database, with the search period from database inception to June 4, 2026. Two researchers screened, organized, and summarized the literature based on inclusion and exclusion criteria.

**Results:**

A total of 22 articles were included. The experiences mainly involved impaired social function, psychological distress, and a lack of support resources; influencing factors were divided into four aspects: demographic characteristics, disease factors, psychological factors, and family and social factors; intervention measures included narrative care, acceptance and commitment therapy, health education, and other content.

**Conclusion:**

The social isolation status of patients with intestinal stomas is influenced by multiple factors. In the future, localized assessment tools should be developed to accurately identify the level of social isolation, helping patients return to society as soon as possible.

## 1 Introduction

Colorectal cancer is a common malignant tumor of the digestive system. It is projected that by 2040, the global burden of colorectal cancer will increase by 63%, with approximately 3.2 million new cases (Morgan et al., 2023). At present, radical resection is the mainstay of treatment for colorectal cancer. However, due to the influence of tumor lesion location and depth of invasion, anus preservation cannot be achieved in some patients, who therefore require enterostomy (Chen et al., 2026). This surgical procedure involves exteriorizing a segment of the bowel, everting the stoma, and suturing it to the abdominal wall to create an artificial anus for facilitating defecation. However, patients after enterostomy face postoperative challenges such as body image disturbance, altered excretory patterns, and stoma-related complications, which in turn lead to adverse psychological outcomes including social isolation, stigma, anxiety, and depression (Ayaz-Alkaya, 2019). Social isolation refers to the estrangement of patients from others and social relationships during social interactions, including being isolated or excluded by others, which triggers negative emotions such as loneliness and helplessness, and leads to passive behaviors such as social avoidance (Liang et al., 2022; Wang and Yang, 2026), seriously affecting patients' mental health. Liao et al. (2024) found that only 13.9% of patients with colorectal cancer ostomy reported low levels of isolation, 64.0% experienced moderate isolation, and 22.1% suffered from the dual burden of high isolation and low sense of meaning. Domestic studies have also shown that the level of social isolation among ostomy patients is moderate or above (Shao et al., 2022). Therefore, it is particularly important to pay attention to the subjective experience, influencing factors, and intervention strategies of social isolation. However, existing studies are mostly limited to a single dimension and lack a systematic integration of the relevant literature in this field. A scoping review can identify the progress in a research field, summarize and organize key content, thereby promoting the development of the knowledge system in this field (Wang et al., 2019). In light of this, this study employed the Arksey and O'Malley scoping review framework (Qiu and Gu, 2022), to systematically identify and synthesize the subjective experiential characteristics of social isolation among patients with intestinal stomas, map the associated influencing factors and intervention strategies, and provide a reference for subsequent clinical research.

## 2 Materials and methods

### 2.1 Identifying the research questions

① What are the psychological experiences of social isolation among patients with intestinal stomas? ② What factors influence social isolation among patients with intestinal stomas? ③ What interventions have been developed to address social isolation in patients with intestinal stomas, and what are their effectiveness?

### 2.2 Search strategy

A systematic search was conducted from database inception to June 4, 2026, using a combination of subject headings and free-text keywords in both Chinese and English databases. The following databases were searched: CNKI, Wanfang Data, VIP Database, SinoMed, PubMed, Web of Science, Embase, Cochrane Library, and CINAHL. The search terms were: “ostomy/enterostomy/ostomies/intestinal ostomy/neostomy/stoma/enterostomies/intestinal fistula/intestinal stomas/ileostomy /colostomy” “social isolation /social isolat^*^/ social alienation^*^/loneliness/social exclusion^*^”.

### 2.3 Inclusion criteria

① Participants were adults aged ≥18 years with intestinal stomas; ② Studies addressed social isolation or social alienation among patients with intestinal stomas; ③ Study designs included qualitative studies, cross-sectional studies, longitudinal studies, quasi-experimental studies, and randomized controlled trials (RCTs).

### 2.4 Exclusion criteria

Reviews, systematic reviews, conference abstracts, experience-sharing articles, and dissertations/theses; Studies published in languages other than Chinese or English; Studies for which the full text could not be obtained.

### 2.5 Study selection and data extraction

Two researchers independently screened the literature and extracted data according to the inclusion and exclusion criteria. Any disagreements were resolved by a third researcher. The extracted data included: author, publication year, country, study design, sample size, participants, experiences, prevalence/severity, influencing factors, outcome measures, and assessment tools.

## 3 Results

### 3.1 Literature search results

**Figure 1:**
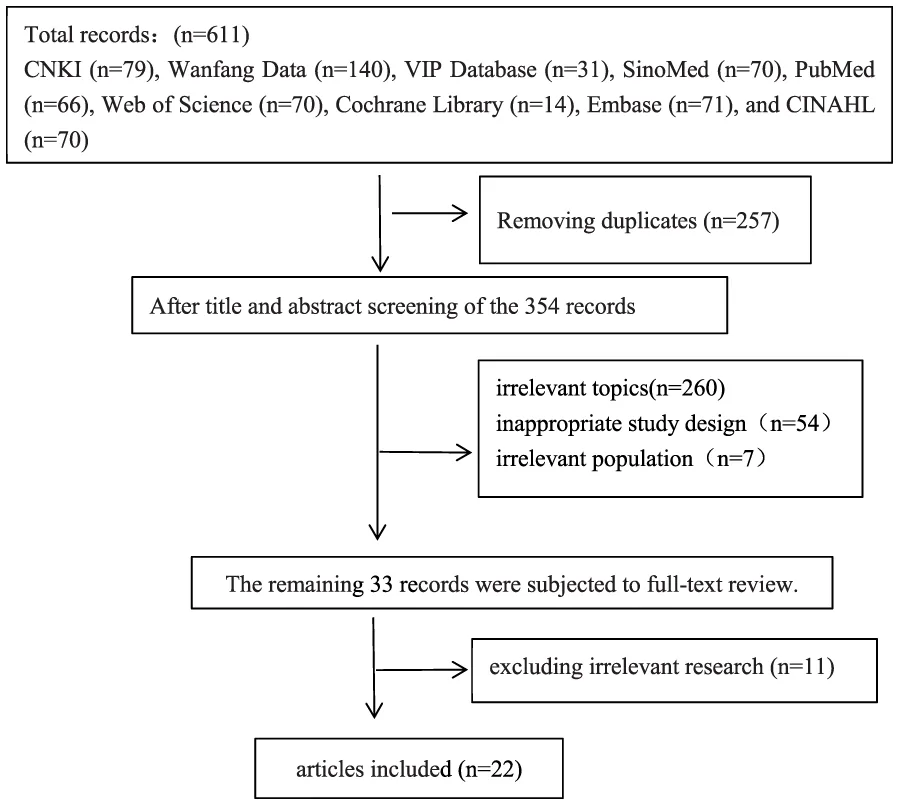
Flow chart of literature screening.

A total of 611 records were initially retrieved. After deduplication, 354 records remained. Of these, 321 were excluded after title and abstract screening, and an additional 11 were excluded following full-text review. Ultimately, 22 studies were included. The screening process is illustrated in Figure 1.

### 3.2 Basic characteristics of the included studies

A total of 22 studies were included in this review (Duanmu et al., 2022; Fan et al., 2025; Gao et al., 2025; Hu et al., 2026; Ji et al., 2023; Shao et al., 2022; Liao et al., 2024; Liu et al., 2024; Mathieu et al., 2025; Wang, 2022; Wang et al., 2017, 2026; Wu, 2023; Xu et al., 2024, 2025; Xue et al., 2021; Yang et al., 2023; Zhang and Qu, 2026; Zhang M. J. et al., 2025; Zhao et al., 2024; Zhao and Yao, 2024), among which 3 were qualitative research (Mathieu et al., 2025; Xu et al., 2024; Yang et al., 2023), two were cohort studies (Xu et al., 2025; Hu et al., 2026), 11 were cross-sectional studies (Duanmu et al., 2022; Fan et al., 2025; Shao et al., 2022; Liao et al., 2024; Liu et al., 2024; Wang et al., 2017; Wu, 2023; Zhang and Qu, 2026; Zhang M. J. et al., 2025; Zhao and Yao, 2024; Zhao et al., 2024), and six were interventional studies. The detailed information is presented in Tables 1–3.

**Table 1:** Basic characteristics of literature on the subjective experience of social isolation in patients with enterostomy.

Study	Publication year	Country	Study design	Sample size	Participants	Experience
Xu et al. (2024)	2024	China	Qualitative research	19	Colorectal cancer stoma patients	Impaired social competence; Insufficient social motivation; Imperfect social support system
Yang et al. ((2023)	2023	China	Qualitative research	12	Permanent enterostomy patients	Subjective experiences of social isolation; Behavioral manifestations of social alienation; Contributing factors to social alienation; Patients' needs
Mathieu et al. (2025)	2025	Switzerland	Qualitative research	9	Permanent colostomy patients	Restricted activities leading to social alienation; Disturbed body image with prominent demand for psychological support; Insufficient stoma education and follow-up care; Contradiction between willingness to seek support and concealment of stoma condition

**Table 2:** Basic characteristics of studies on factors influencing social isolation in patients with enterostomy.

Study	Publication year	Country	Study design	Sample size	Participants	Incidence and severity	Assessment tool	Influencing factors
Liao et al. (2024)	2024	China	Cross-sectional study	303	Colorectal cancer stoma patients	Low alienation group (13.9%); Moderate alienation group (64.0%); High alienation-low meaningness group (22.1%)	GAS	Educational level, occupation, household economic income, weekly frequency of ostomy bag replacement
Shao et al. (2022)	2022	China	Cross-sectional study	319	Colorectal cancer patients with stoma	Upper-moderate level	GAS	Age, postoperative ostomy duration, occupation-al status, personality type, participation in stoma mutual support group, stomy self-care ability, psychological vulnerability, social support
Xu et al. (2025)	2025	China	Cohort study	232	Young and middle-aged patients with enterostomy	High social isolation-slow decline trajectory (16.4%); Moderate social isolation-rapid decline trajectory (45.4%); Low social isolation-stable trajectory (38.2%)	SIS-C	Temperament type, social influence
Hu et al. (2026)	2026	China	Cohort study	179	Permanent colostomy patients after rectal cancer surgery	High social isolation-slow decline group (30.17%); Moderate social isolation-rapid decline group (46.93%); Low social isolation-stable group (22.91%)	SIS-C	Age, intestinal stoma acceptance, participation in stoma fellowship activities, stoma self-care ability, anxiety, depression, family care index
Wu (2023)	2023	China	Cross-sectional study	302	Rectal cancer stoma patients	Moderate level	GAS	Age, stigma, stoma acceptance, stoma care status, stoma complications
Duanmu et al. (2022)	2022	China	Cross-sectional study	242	Elderly stoma patients	Relatively high level	GAS	Gender, education level, postoperative ostomy duration, social participation, social relationship quality, activities of daily living
Zhao et al. (2024)	2024	China	Cross-sectional study	110	Permanent colostomy patients	Moderate level	IAS	Postoperative ostomy duration, annual hospitalization frequency, stoma self-care ability, coping strategies, stigma, perceived family support
Zhang M. J. et al. (2025)	2025	China	Cross-sectional study	304	Colorectal cancer stoma patients	Upper-moderate level	GAS	Medical payment method, medical coping style
Fan et al. (2025)	2025	China	Cross-sectional study	592	Colorectal cancer patients with temporary stoma	Low avoidance-low distress group (40%); moderate avoidance-low distress group (37.2%);high avoidance-high distress group (22.8%)	SADS	Gender, age, awareness of surgical plan, stoma self-care ability, stoma outpatient visit status self-efficacy, stigma
Wang et al. (2017)	2017	China	Cross-sectional study	228	Permanent colostomy	Relatively high level	SADS	Gender, occupation, postoperative ostomy duration, stigma, self-esteem, self-concealment
Zhao and Yao (2024)	2024	China	Cross-sectional study	240	Colorectal cancer patients with permanent stoma	Relatively high level	GAS	Gender, age, household economic income, disease-related factors, depression symptoms, level of social support, stigma
Liu et al. (2024)	2024	China	Cross-sectional study	267	Colorectal cancer patients with stoma	High level	SIS-C	Gender, age, postoperative ostomy duration, stoma self-care ability, stoma complications, postoperative chemoradiotherapy status, perceived discrimination
Zhang and Qu (2026)	2026	China	Cross-sectional study	507	Colorectal cancer patients with stoma	41.6%	GAS	Gender, place of residence, stoma type, stigma, social support, psychological vulnerability, stoma acceptance

SIS-C, social isolation scale for colorectal cancer survivor; GAS, general alienation scale, IAS, interaction anxiousness scale, SADS or SAD, social avoidance and distress scale.

**Table 3:** Basic characteristics of studies on social isolation interventions in patients with enterostomy.

Study	Publication year	Country	Study design	Sample size (intervention group/control group, *n*)	Participants	Intervention	Intervention duration	Outcome measures	Assessment tools
Xue et al. (2021)	2021	China	Quasi-experimental study	51	Young and middle-aged patients with permanent stoma	Narrative therapy	3 weeks	Post-traum-atic stress level, self-manageme-nt ability, social avoidance	PCL-C, OP-SMS, SADS
Gao et al. (2025)	2025	China	RCT	47/45	Permanent stoma patients	Control group: routine nursing; Interventiongroup: routine nursing+acceptance and commitment therapy	1 month	Social avoidance, stoma acceptance, ostomy complicati-ons incidence	SAD, SAQ
Ji et al. (2023)	2023	China	RCT	54/54	permanent stoma patients	Control group: routine health education, Intervention group: routine health education + family empowerme-nt intervention	8 weeks	social alienation, perceived personal life control, ostomy complicati-ons incidence	GAS, PMA
Wang et al. (2026)	2026	China	RCT	36/36	Rectal cancer patients with stoma	Control group: routine nursing; Intervention group: routine nursing + ABC theory of Emotion-based supportive care intervention	3 months	Social alienation, perceived discriminat-ion level, loneliness level	SIS-C, SIS, loneliness scale
Wang (2022)	2022	China	RCT	40/40	Stoma patients	Control group: routine health education; Intervention group: precede-proceed model-based health education	8 weeks	Social avoidance, stoma self-care ability, ostomy complicati-ons incidence	SADS, SSCS-GV
Zhang M. J. et al. (2025)	2025	China	RCT	41/41	Stoma patients	Control group: health education; intervention group: LEARNS model-based health education	-	Health knowledge mastery, self-care ability, quality of life, social alienation, ostomy complicati-ons incidence	Self-develo-ped enterostom-y health knowledge assessment scale, ESCA, SF-36, GAS

PCL-C, the PTSD checklist-civilian version; OP-SMS, ostomy patients self-management scale; SAQ, stoma acceptance questionnaire; PMS, personal mastery scale; SIS, social impact scale; SSCS-GV, stoma self-care scalegeneral version; ESCA, exercise of self-care agency; SF-36, medical outcomes study short-form 36 health survey; SSES, stoma self-efficacy scale.

### 3.3 Psychological experience of social alienation among intestinal stoma patients

Three qualitative studies (Mathieu et al., 2025; Xu et al., 2024; Yang et al., 2023) indicated that social alienation among intestinal stoma patients was externally manifested as impaired social functioning and avoidance of social behaviors, accompanied by internalized negative psychological experiences such as low self-esteem, loneliness, and stigma. The alienation experience was collaboratively induced by multidimensional factors encompassing individual psychology, physical condition, and social environment. Xu et al. (2024) identified three themes: physical complications, activity restrictions, and excessive caregiving burden led to impaired social functioning; stigma, sense of meaninglessness, and lack of security further reduced social initiative; meanwhile, family dysfunction and public misconceptions about the disease resulted in a deficient social support system, thereby exacerbating the process of social alienation among patients. Yang et al. (2023) summarized patients' psychological experiences from four perspectives: subjective feelings, external manifestations, contributing factors, and health needs. At the subjective level, patients experienced negative emotions such as loneliness, low self-esteem, and stigma. Externally, their behaviors were characterized by social avoidance, changes in social circles, and altered interests and hobbies. Self-stigma, social discrimination, multiple social role conflicts, and a lack of social support resources were identified as key factors contributing to social alienation. Meanwhile, disseminating stoma-related health knowledge, expanding channels for psychological intervention, and returning to work emerged as the primary demands of patients. Mathieu et al. (2025) pointed out that patients with permanent colostomy in Switzerland generally face multiple adaptation dilemmas, including physical activity limitation, impaired body image, insufficient stoma health education, deficient out-of-hospital follow-up management, and concealment of stoma-related conditions. These various problems interact and overlap with each other, which further intensifies the patients' experience of social alienation. In summary, social alienation among intestinal stoma patients results from the coupling of multiple factors, including physiological trauma, negative self-cognition, social prejudice, and insufficient support resources, forming a compound alienation dilemma with physiological, psychological, and social interactions.

### 3.4 Factors influencing social alienation among intestinal stoma patients

#### 3.4.1 Demographic factors

① Age: Six studies (Fan et al., 2025; Hu et al., 2026; Shao et al., 2022; Liu et al., 2024; Wu, 2023; Zhao and Yao, 2024) consistently confirmed that age is one of the key factors influencing social alienation among intestinal stoma patients. Hu et al. (2026) found that patients under 60 years of age exhibited higher levels of social alienation. Zhao et al. (2024) echoed this view, noting that permanent colorectal cancer stoma patients aged 18–44 years experienced a higher degree of social alienation. Additionally, two studies (Shao et al., 2022; Wu, 2023) found that younger age was a contributing factor to the development of social alienation among intestinal stoma patients. ② Gender: Six studies (Duanmu et al., 2022; Fan et al., 2025; Liu et al., 2024; Wang et al., 2017; Zhang and Qu, 2026; Zhao and Yao, 2024) consistently found that female patients were more prone to social avoidance behaviors. ③ Education level: two studies (Liao et al., 2024; Wu, 2023) indicated that colorectal cancer patients with lower education levels exhibited relatively higher levels of social alienation. ④ Employment status: Employed individuals exhibited lower levels of social alienation (Shao et al., 2022; Liao et al., 2024), whereas patients with farming occupations showed relatively higher levels of social alienation (Wang et al., 2017). ⑤ Personality traits: Patients with extroverted personalities exhibited lower levels of social alienation compared to those with introverted personalities (Shao et al., 2022), while patients with depressive personality types showed higher levels of social alienation (Xu et al., 2025). ⑥ Economic status: Patients with lower economic status exhibited higher levels of social alienation (Liao et al., 2024; Zhao and Yao, 2024). ⑦ Patients who paid out-of-pocket or through commercial insurance exhibited higher levels of social alienation (Zhang M. J. et al., 2025). ⑧ Residence: The risk of social alienation among rural patients is higher than that among urban patients (Zhang and Qu, 2026).

#### 3.4.2 Disease factors

Stoma patients with the following disease-related factors exhibited higher levels of social isolation: primarily including low stoma self-efficacy (Fan et al., 2025), low stoma self-care ability (Fan et al., 2025; Hu et al., 2026; Liu et al., 2024; Zhao et al., 2024), dependence on others for stoma care (Shao et al., 2022; Wu, 2023), short time since stoma surgery (Duanmu et al., 2022; Shao et al., 2022; Liu et al., 2024; Wang et al., 2017; Zhao et al., 2024), permanent stoma type (Zhang and Qu, 2026), stoma-related complications (Liu et al., 2024; Wu, 2023), significant stoma-related symptom burden (Zhao and Yao, 2024), frequent hospitalizations (Zhao et al., 2024), undergoing chemotherapy or radiotherapy (Liu et al., 2024), avoidant or resigned coping styles (Zhang M. J. et al., 2025), unawareness of the surgical plan (Fan et al., 2025), and regular outpatient follow-up visits (Fan et al., 2025), the frequency of weekly stoma replacement is ≤ 2 times (Liao et al., 2024).

#### 3.4.3 Psychological factors

Stoma patients with the following psychological factors exhibited higher levels of social isolation: low stoma acceptance (Hu et al., 2026; Zhang and Qu, 2026), high stigma (Fan et al., 2025; Wu, 2023; Wang et al., 2017; Xu et al., 2025; Zhang and Qu, 2026; Zhao and Yao, 2024; Zhao et al., 2024), anxiety (Hu et al., 2026), depression (Hu et al., 2026; Zhao and Yao, 2024), higher levels of perceived discrimination (Liu et al., 2024), higher levels of self-concealment (Wang et al., 2017), low self-esteem (Wang et al., 2017), and higher psychological vulnerability (Shao et al., 2022; Zhang and Qu, 2026).

#### 3.4.4 Family and social factors

Stoma patients with the following family and social factors exhibited higher levels of social isolation: moderate family care (Hu et al., 2026; Zhao et al., 2024), infrequent participation in stoma association activities (Hu et al., 2026; Shao et al., 2022), low levels of social participation (Duanmu et al., 2022), low quality of social relationships (Duanmu et al., 2022), and low levels of social support (Shao et al., 2022; Zhang and Qu, 2026; Zhao and Yao, 2024).

### 3.5 Interventions for social isolation in stoma patients

Regarding social isolation among stoma patients, the effectiveness of interventions has been validated in related studies. Xue et al. (2021) implemented six sessions of narrative therapy for young and middle-aged patients with permanent stomas, using the SADS scale to assess scores before and after the intervention. The results showed that social avoidance behaviors were significantly lower after the intervention compared to baseline. The underlying mechanism involves externalization, rewriting, reshaping dialogue, and defining ceremonies, which separate patients from negative emotions, correct cognitive biases, explore positive events, and provide social support, thereby reducing social avoidance and enhancing self-management levels. Gao et al. (2025) implemented six sessions of acceptance and commitment therapy for patients with permanent stomas, and the results showed that the intervention group had significantly lower SAD scores compared to the control group. The underlying mechanism is as follows: through acceptance and cognitive defusion, patients are guided to face the reality of their stoma and distinguish thoughts from reality; combined with mindfulness practice to experience the present moment, and relying on values clarification and action planning, stigma and social fear are gradually dissolved, and stoma acceptance is enhanced. Ji et al. (2023) conducted an 8-week family empowerment intervention for patients with permanent stomas. Between-group comparisons revealed that the observation group had significantly lower scores across all GAS dimensions compared to the control group. This intervention model targeted caregivers with in-hospital cognitive interventions, provided online guidance for patients and caregivers on stoma bag replacement, delivered complication prevention and control knowledge through graphic and video materials, and established a communication channel *via* the WeChat platform. By taking caregivers as the entry point and leveraging the advantages of stoma transitional care, this intervention aims to reduce the level of social isolation. Wang et al. (2026) confirmed that supportive care therapy based on the ABC theory of emotions could improve social isolation in stoma patients. This therapy, with the ABC theory of emotions as its core framework, first assesses and identifies triggering factors of social isolation, then corrects misconceptions about stomas, while monitoring behavioral indicators such as social withdrawal. Furthermore, it delivers comprehensive and systematic supportive care interventions centered on four core needs: physiological, informational, psychological, and social. Wang (2022) conducted an RCT involving 80 stoma patients. The control group received routine health education, while the observation group received health education based on the Green model on top of routine care. The results showed that the observation group had significantly lower SADS scores compared to the control group. The intervention mechanism was based on the Green model, implementing interventions across three factors: predisposing, enabling, and reinforcing. Predisposing factors: stoma knowledge was disseminated through a smart platform, and patients were encouraged to participate in social activities. Enabling factors: targeted communication was conducted according to patients' cognitive levels, guiding them to express their inner emotions. Reinforcing factors: family and social support resources were integrated to facilitate patients' return to society. Zhang X. Z. et al. (2025) applied the LEARNS model of health education to stoma patients, delivering systematic interventions through six steps: listening, explaining, acknowledging, recommending, negotiating, and summarizing. The GAS scale was used to assess patients' social isolation. The results showed that the observation group had significantly lower social isolation scores compared to the control group. The underlying mechanism involves first assessing and listening to patients' physical and psychological conditions and actual needs, followed by explaining the significance of health education under the LEARNS model. Subsequently, stoma-related knowledge and skills were disseminated, followed by question-and-discussion sessions. The education plan was optimized based on feedback, supplemented by long-term follow-up supervision, aiming to provide personalized and diversified health education.

## 4 Discussion

### 4.1 Practical value of social alienation level and its influencing factors in patients with intestinal stomas

The practical value of research on the social alienation level and its influencing factors among intestinal stoma patients is mainly reflected in three aspects. First, high-risk populations can be identified based on the level of social alienation, so as to optimize the clinical risk screening practice. Four studies have confirmed that patients with permanent intestinal stomas experience significant social alienation (Hu et al., 2026; Wang et al., 2017; Zhao and Yao, 2024; Zhao et al., 2024). Zhang and Qu (2026) pointed out that permanent stoma is an independent risk factor for social alienation. Xu et al. (2025) found that the problem of social alienation is more prominent among young and middle-aged intestinal stoma patients. A study by Duanmu et al. (2022) showed that elderly intestinal stoma patients are at a relatively high level of social alienation. In the future, patients with permanent stoma, young and middle-aged intestinal stoma patients, and elderly intestinal stoma patients should be clinically identified as high-risk groups and provided with standardized screening and management. However, the current assessment tools for social alienation are not unified. Only three studies (Hu et al., 2026; Liu et al., 2024; Xu et al., 2025) adopted the Supports Intensity Scale—Children's Version (SIS-C) for assessment. This scale is developed based on the theoretical framework of the social alienation conceptual model and has certain specificity. Subsequent research needs to expand the geographical scope to verify its reliability and validity. Second, studies have shown that due to the influence of regional factors and cultural concepts, the social avoidance behavior of stoma patients in China is significantly higher than that in Nordic countries (Kristensen et al., 2022). There is an urgent need to develop localized assessment tools, aiming to accurately identify the social alienation level of patients. Third, clinically, an individualized comprehensive intervention program can be constructed based on multi-dimensional influencing factors. At the demographic characteristic level, for low-income groups, relevant information such as medical insurance reimbursement policies and affordable stoma care supplies should be actively popularized; for rural stoma patients, tele-nursing channels should be built, and regular online specialized stoma guidance should be carried out; attention should be paid to the body image anxiety of young and middle-aged women, and emotional counseling should be provided for them. At the disease management level, departments should strengthen stoma skill training and carry out practical teaching. Existing studies have shown that targeted stoma skill training for patients can significantly improve their stoma self-care ability (Ko et al., 2023). Meanwhile, Ning et al. (2022) found that the relevant caregivers of stoma patients also face a high level of loneliness. Subsequent studies should also take the dual disease management mechanism of patients and their caregivers as a breakthrough to carry out multi-center, large-sample prospective studies.

### 4.2 The breakthroughs and advantages of this study compared with previous related research

Traditional review studies on social alienation in patients with intestinal stomas mostly conduct simple induction and sorting on the concepts, influencing factors, assessment tools, and intervention measures of social alienation (Ji et al., 2022; Li et al., 2024; Qiao et al., 2024). The literature integration is highly subjective, while the retrieval strategies and inclusion-exclusion criteria lack transparency, which separates the results of quantitative data and qualitative studies. In addition, the two included systematic reviews have a single type of literature, which can only explain the subjective experience of social alienation without the support of objective data (Lin et al., 2026; Zhang et al., 2024). This study is the first to adopt the scoping review method to conduct systematic retrieval and information integration of relevant studies on social alienation in intestinal stoma patients. It deeply excavates the subjective experience of social alienation and the evolution trajectory of psychological adaptation of intestinal stoma patients in qualitative studies; based on cohort studies and cross-sectional studies, it quantifies the current status and incidence of social alienation, and refines the classification of its influencing factors; it extracts the mechanism of action, outcome indicators, assessment tools and intervention effects of each intervention measure from quasi-experimental studies and RCTs. The above core research results are presented in the form of structured tables, which are intuitive, clear and highlight key points, making it convenient for clinical medical staff to quickly capture key information. Secondly, this study identifies the gaps in the current field from two dimensions: study types and publishing countries. In terms of study types, only two foreign studies involve qualitative research and cross-sectional surveys (Mathieu et al., 2025; Zhang and Qu, 2026), and there is a lack of prospective, large-sample intervention empirical studies; most domestic studies focus on status surveys and intervention research. From the perspective of publishing countries, China accounts for a relatively high proportion of publications in this field, while abroad, only Switzerland has conducted qualitative interviews with patients with permanent intestinal stomas to explore their adaptation dilemmas during the transition period. In the future, targeting the limitation of unbalanced regional research distribution, comparative studies on social alienation among intestinal stoma patients at home and abroad should be carried out to explore the moderating effects of cultural values, medical security systems and family care models on the formation mechanism of social alienation. Meanwhile, mixed research methods should be adopted to reveal the core driving factors of social alienation in intestinal stoma patients and the breakthrough points for intervention.

## 5 Conclusion

This study summarized the subjective experiences, influencing factors, and interventions of social isolation among stoma patients. However, the included literature is predominantly in Chinese, and no quality assessment of the studies was conducted, which may introduce bias into the conclusions. Existing research has focused predominantly on the influencing factors of social isolation in stoma patients, while qualitative and interventional studies remain relatively scarce, and the research framework is not yet well-established. Future research should conduct long-term longitudinal follow-up across different stoma types to systematically analyze the dynamic changes in patients' social isolation. Meanwhile, multidisciplinary collaborative teams should be established to carry out large-sample interventional studies, thereby effectively improving the social isolation status of stoma patients and facilitating their early return to society.
