# Supplementary material for: A scoping review on social isolation in patients with intestinal stomas
Source: Front Psychol. 2026 Aug 19;17:1919034. doi: 10.3389/fpsyg.2026.1919034 (PMC13535527; doi:10.3389/fpsyg.2026.1919034)
Supplement: Supplementary file 1 [file Table_1.docx]

Retrieval strategies for major databases

1.PubMed

| serial number | ‌search term‌ |
| --- | --- |
| #1 | ostomy[MeSH Terms] OR enterostomy[MeSH Terms] OR ostomies[Title/Abstract] OR intestinal ostomy[Title/Abstract] OR neostomy[Title/Abstract] OR stoma[Title/Abstract] OR enterostomies[Title/Abstract] OR intestinal fistula[Title/Abstract] OR intestinal stomas[Title/Abstract] OR ileostomy[Title/Abstract] OR colostomy[Title/Abstract] |
| #2 | social isolation[MeSH Terms] OR social isolat*[Title/Abstract] OR social alienation*[Title/Abstract] OR loneliness[Title/Abstract] OR social exclusion*[Title/Abstract] |
| #3 | #1AND#2 |

2.Web of Science

| serial number | ‌search term‌ |
| --- | --- |
| #1 | Topic:ostomy OR enterostomy OR ostomies OR intestinal ostomy OR neostomy OR stoma OR enterostomies OR intestinal fistula OR intestinal stomas OR ileostomy OR colostomy |
| #2 | Topic:social isolation OR social isolat* OR social alienation* OR loneliness OR social exclusion* |
| #3 | #1AND#2 |

3.Cochrane Library

| serial number | ‌search term |
| --- | --- |
| #1 | (Ostomy）:ti,ab,kw OR (enterostomy):ti,ab,kw OR (ostomies):ti,ab,kw OR (intestinal ostomy):ti,ab,kw OR (neostomy):ti,ab,kw OR (stoma):ti,ab,kw OR (enterostomies):ti,ab,kw OR (intestinal fistula):ti,ab,kw OR (intestinal stomas):ti,ab,kw OR (ileostomy) :ti,ab,kwOR (colostomy):ti,ab,kw |
| #2 | (social isolation) :ti,ab,kw OR (social isolat*) :ti,ab,kw OR (social alienation*) :ti,ab,kw OR (loneliness) :ti,ab,kw OR (social exclusion*):ti,ab,kw |
| #3 | #1AND#2 |

4.Embase

| serial number‌ | ‌search term |
| --- | --- |
| #1 | (ostomy):ti,ab,kw OR (enterostomy):ti,ab,kw OR (ostomies):ti,ab,kw OR (intestinal ostomy):ti,ab,kw OR (neostomy):ti,ab,kw OR (stoma):ti,ab,kw OR (enterostomies):ti,ab,kw OR (intestinal fistula):ti,ab,kw OR (intestinal stomas):ti,ab,kw OR (ileostomy):ti,ab,kw OR (colostomy):ti,ab,kw |
| #2 | (social isolation) :ti,ab,kw OR (social isolat*) :ti,ab,kw OR (social alienation*) :ti,ab,kw OR (loneliness) :ti,ab,kw OR (social exclusion*):ti,ab,kw |
| #3 | #1AND#2 |
